# Etiology of recurrent cystitis in postmenopausal women based on vaginal microbiota and the role of *Lactobacillus* vaginal suppository

**DOI:** 10.3389/fmicb.2023.1187479

**Published:** 2023-05-18

**Authors:** Takanori Sekito, Koichiro Wada, Ayano Ishii, Takehiro Iwata, Takehiro Matsubara, Shuta Tomida, Masami Watanabe, Motoo Araki, Takuya Sadahira

**Affiliations:** ^1^Department of Urology, Okayama University Graduate School of Medicine, Dentistry and Pharmaceutical Science, Okayama, Japan; ^2^Department of Urology, Shimane University Faculty of Medicine, Izumo, Shimane, Japan; ^3^Okayama University Hospital Biobank, Okayama University Hospital, Okayama, Japan; ^4^Center for Comprehensive Genomic Medicine, Okayama University Hospital, Okayama, Japan

**Keywords:** cystitis, vagina, microbiota, *Lactobacillus*, urinary tract infection

## Abstract

**Background:**

The vaginal microbiota can be altered by uropathogenic bacteria associated with recurrent cystitis (RC), and the vaginal administration of *Lactobacillus* have suggested certain effects to prevent RC. The relationship between vaginal microbiota and the development of RC has not been elucidated. We aimed to clarify the etiology of RC from vaginal microbiota and importance of vaginal *Lactobacillus*.

**Methods:**

Vaginal samples obtained from 39 postmenopausal women were classified into four groups: healthy controls; uncomplicated cystitis; RC; and prevention (prevented RC by *Lactobacillus crispatus*-containing vaginal suppositories). Principal coordinate analysis and beta-diversity analysis was used to assess 16S rRNA gene sequencing data from the vaginal microbiome.

**Results:**

Cluster analysis divided the vaginal bacterial communities among 129 vaginal samples into three clusters (A, B, and C). Fourteen of 14 (100%) samples from the RC group and 51 of 53 (96%) samples from the prevention group were in clusters B and C, while 29 of 38 (76%) samples from the healthy group and 14 of 24 (58%) samples from the uncomplicated cystitis group were in cluster A. The principal coordinate analysis showed that plots in the uncomplicated cystitis group were similar to the healthy group, indicating a large separation between the RC group and the uncomplicated cystitis group. On beta-diversity analysis, there were significant differences between the healthy group and the uncomplicated cystitis group (*p* = 0.045), and between the RC group and the uncomplicated cystitis group or the healthy group (*p* = 0.001, *p* = 0.001, respectively). There were no significant differences between the RC group and the prevention group (*p* = 0.446). The top six taxa were as follows: *Prevotella, Lactobacillus, Streptococcus, Enterobacteriaceae, Anaerococcus,* and *Bifidobacterium*. Among patients with RC, *Lactobacillus* was undetectable before administration of suppositories, while the median relative abundance of *Lactobacillus* was 19% during administration of suppositories (*p* = 0.0211), reducing the average cystitis episodes per year (6.3 vs. 2.4, *p* = 0.0015).

**Conclusion:**

The vaginal microbiota of postmenopausal women with RC is differed from healthy controls and uncomplicated cystitis in terms of lack of *Lactobacillus* and relatively dominant of *Enterobacteriaceae*. Vaginal administration of *Lactobacillus*-containing suppositories can prevent RC by stabilizing vaginal dysbiosis and causing a loss of pathogenic bacteria virulence.

## Introduction

Urinary tract infections (UTIs) are highly prevalent in women, and recurrent cystitis (RC) can be burdensome in postmenopausal women (Jung and Brubaker, 2019). It was originally suggested that bacteria colonizing the vagina enter the urinary tract due to proximity (Beerepoot and Geerlings, 2016). The vagina can serve as a reservoir of enteric bacteria and vaginal dysbiosis characterized by a decrease of *Lactobacilli* and colonization with *Escherichia coli* (*E. coli*) in the vaginal microbiota can lead to RC (Pabich et al., 2003; Muhleisen and Herbst-Kralovetz, 2016; Stapleton, 2016). Although the current guidelines recommend antibacterial prophylaxis for women with recurrent UTIs (Anger et al., 2019; Bonkat et al., 2019), frequent and long-term use of antimicrobial agents leads to intractable cystitis (Gupta and Bhadelia, 2014). We previously showed that RC can be caused by vaginal dysbiosis and found that changes in the vaginal microbiota during the administration of *Lactobacillus crispatus* (*L. crispatus*)-containing vaginal suppositories prevents RC, and even refractory RC (Uehara et al., 2006; Sadahira et al., 2021). In the phase II clinical trial, 21 women completed the study, and a significant reduction in the mean number of episodes of cystitis before and during administration of *Lactobacillus*-containing vaginal suppositories was observed using Mann–Whitney *U*-test (Before treatment, 5.62 episodes; During treatment, 2.52 episodes [*p* = 0.00007]) without administration-related adverse events (Sadahira et al., 2021). Other clinical trials reported that some vaginal probiotics, such as vaginal *Lactobacillus*-containing suppositories and intravaginal estriol, are associated with preventing RC by affecting the vaginal microbiota (Raz and Stamm, 1993; Kwok et al., 2006; Stapleton et al., 2011; Ferrante et al., 2021). In a recent double-blind, placebo-controlled phase 2 study, Stapleton et al. investigated 100 women with RC. The patients were divided into two groups, and were given a placebo or probiotics (Lactin-V) containing *L. crispatus* (10^8^ CFUs/mL) intravaginally for 10 weeks. Recurrent UTI (rUTI) occurred in only 15% of the patients with the probiotic treatment compared to 27% with the placebo treatment significantly (relative risk, 0.5; 95% confidence interval, 0.2–1.2; Stapleton et al., 2011). Raz et al. reported that intravaginal estriol therapy reduced the incidence of rUTI with increasing vaginal *Lactobacillus* populations (0.5 vs. 5.9 episodes per patient-year, *p* < 0.001) (Raz and Stamm, 1993), and one recent randomized clinical trial demonstrated a significant reduction in the incidence of rUTI among postmenopausal women using intravaginal estrogen compared with placebo (8/15 vs. 10/11, respectively; *p* = 0.036; Ferrante et al., 2021).

Recently, molecular techniques using 16S ribosomal ribonucleic acid (rRNA)-based gene sequencing analysis have become more affordable and accessible to profile the composition of the microbiome (van de Wijgert et al., 2014). Studies using 16S rRNA sequencing analysis recently reported that the vaginal microbiota in women is dominated by *Lactobacilli*, including *L. crispatus* (Smidt et al., 2015; Lee et al., 2020; Sirichoat et al., 2021; Hugenholtz et al., 2022). In one report, changes in the vaginal microbiota by decreasing *Lactobacillus* species and increasing the diversity of organisms were observed in postmenopausal women with RC compared with premenopausal women, showing postmenopausal status has a strong impact on microbiome composition (Hugenholtz et al., 2022). Vaginal microbiota has been considered as a key factor for increasing the risk of UTI in current literatures (Lewis and Gilbert, 2020), however, analyses comparing the vaginal microbiota in postmenopausal women with RC and uncomplicated cystitis compared to healthy postmenopausal women, as well as changes in the vaginal microbiota before and during intervention with *Lactobacillus*-containing vaginal suppositories in postmenopausal women with RC, have been poorly reported.

We conducted this study to demonstrate that the vaginal microbiota is associated with the pathogenesis of RC by comparing the vaginal microbiota among healthy postmenopausal women to postmenopausal women with uncomplicated cystitis and RC using 16S rRNA sequencing analysis. Moreover, we evaluated bacterial abundance to estimate the bacterial taxa of vaginal communities and the number of episodes of RC before and during administration of *Lactobacillus*-containing vaginal suppositories to determine how the vaginal microbiota is changed by intervention with *Lactobacillus*-containing vaginal suppositories.

## Materials and methods

### Study population and episodes of cystitis

This was a retrospective cohort analysis of data from a longitudinal cohort. Data obtained according to the previously reported protocols are embedded (Sadahira et al., 2021). This study was approved by the Institutional Review Board of Okayama University Graduate School of Medicine, Dentistry, and Pharmaceutical Sciences (Ethical approval number: K2208-073). The study participants reviewed and signed the informed consent document.

We focused on postmenopausal women ≥50 years of age with RC who presented to Okayama University Hospital between December 2013 and October 2020. Patients were excluded for the following reasons: a history of hysterectomy; use of vaginal estrogen suppositories within 1 month prior to study enrollment; required treatment for urologic conditions; ongoing use of an indwelling urethral catheter; a history of allergic hypersensitivity to ingested dairy or *Lactobacillus* products; repeated antibiotic exposure as prophylaxis or treatment for recurrent UTIs over years; lack of vaginal specimens; and poor compliance.

According to the current guidelines, RC is defined as follows: (1) ≥ 3 episodes of acute bacterial cystitis within 1 year requiring treatment with antibiotics; or (2) ≥ 2 episodes of acute bacterial cystitis within 6 months requiring treatment with antibiotics (Anger et al., 2019; Bonkat et al., 2019). Vaginal specimens provided from postmenopausal women were enrolled into four groups: (1) healthy; (2) uncomplicated cystitis; (3) RC; and (4) prevention (Supplementary Figure 1). The healthy participants were enrolled in the healthy group. Patients who had an episode of cystitis requiring treatment with antibiotics in a 1-year period were enrolled in the uncomplicated cystitis group. For participants in the healthy and uncomplicated cystitis groups, 2 vaginal samples were obtained 1 month apart by taking another sample 1 month later from the baseline visit. The vaginal samples from the uncomplicated cystitis group were collected at the time of cystitis. For included patients with RC, some reference samples from patients with RC were taken before suppository initiation and these samples were regarded as samples in the RC group. During prevention, some samples were taken when the patients got the recurrence of RC. At that time, these samples were regarded as the samples in the RC group although these patients were taking vaginal suppository. Patients with a history of RC prevented by administration of *Lactobacillus*-containing vaginal suppositories and with no recurrence of RC when sampling were enrolled in the prevention group. Vaginal samples from patients in the prevention group were obtained during the time vaginal suppositories were placed, and basically, samples were taken at a follow-up or revisit on 1 to 3-month basis during 1 year.

### Vaginal suppositories

We used *L. crispatus*-containing vaginal suppositories (GAI98322), as previously reported (Uehara et al., 2006; Sadahira et al., 2021). The safety of the suppositories was showed in the previous reports, as no treatment-related adverse events were observed. The vaginal suppositories were manufactured following a viability assay by our hospital pharmacy. The study participants with RC were instructed to insert one vaginal suppository before going to bed, either every 2 days or 3 times per week, for 1 year. Patients were instructed how to insert the suppositories in detail, as to carefully push the suppository using their finger until its tip was approximately 1 cm deeper than the vaginal orifice, and to lie still for approximately 20 min until the suppository dissolved. At follow-up visit to the hospital, we got questionnaire to check the usage of the suppositories to guarantee the compliance. If there was evidence of RC, an antimicrobial agent was administered without disrupting the schedule for administration of the vaginal suppositories.

### DNA purification

All vaginal discharge specimens were obtained from participants using cotton swabs (Copan Plain System P; COPAN Diagnostics Inc., Murrieta, California, United States). To obtain vaginal samples, the study participants were placed in the dorsal lithotomy position by a trained urologist in the Outpatient Department. The collected samples were stored at 4°C until extraction was performed. Specifically, the shaft of the cotton swab was cut with scissors, placed in a test tube with 0.4 mL of Dulbecco’s phosphate-buffered saline, and agitated with a vortex mixer for 1 min. DNA was extracted using a QIAamp DNA Mini Kit (Qiagen, Hilden, Germany) from each collected sample following the manufacturer’s instructions.

### Vaginal microbiome analysis

The V3 and V4 regions of the 16S rRNA gene were amplified using 357F (5’-TCGTCGGCAGCGTCAGATGTGTATAAGAGACAGCCTACGGGNGGCWGCAG-3′) and 781R (5’-GTCTCGTGGGCTCGGAGATGTGTATAAGAGACAGGACTACHVGGGTATCTAATCC-3′) primers. The thermal cycling conditions were as follows: 98°C for 3 min; 25 cycles at 98°C for 30 s, 55°C for 30 s, and 72°C for 30 s; and a final extension at 72°C for 5 min. After the first PCR clean-up step using AMpure XP Beads (Beckman Coulter, Inc., Brea, CA, USA), a second PCR was performed to add dual-index barcodes and sequencing adapters to the amplicon target, aiming to distinguish amplicons from each sample by using the same reaction conditions with 8 cycles instead of 25 cycles. After the second PCR clean-up step, the concentration and the degree of amplicon purification were verified using a KAPA library quantification kit (KAPA Biosystems Inc., Wilmington, MA, United States). To form pooling libraries, aliquots (4 nM [5 μL]) of the diluted amplicon from each library were combined. The purified amplicons (4 p/μL) in the pooling libraries were sequenced using the MiSeq® platform (MiSeq Reagent version 3, 600 cycles; Illumina, San Diego, CA, United States) at Okayama University Hospital Biobank (Okayama University Hospital, Okayama, Japan), following the standard protocol. At the time of test setup, 16S quantitative PCR analysis was performed on negative controls and vaginal samples to check the contamination of the DNA extraction process. The number of raw DNA sequence reads assigned to each of the taxa were counted, and the data were cleaned to include only samples with >1,500 reads. The datasets generated for the present study can be found in DRA Accession No. DRA011382 and No. DRA011169.

Principal coordinate analysis (PCoA) was used to characterize and illustrate the clear distinction among vaginal bacterial communities in the four groups. PCoA was performed using the Quantitative Insights Into Microbial Ecology approach (QIIME2) (Hall and Beiko, 2018) for the obtained sequence data to explore and visualize similarities or dissimilarities of data. Hierarchical clustering analysis was performed with the dimensionality reduction data from axes 1–5 of PCoA.

### Statistical analysis

Statistical analyses were performed using EZR software (version 1.71; Saitama Medical Center, Jichi Medical University, Saitama, Japan; Kanda, 2013). Patient characteristics were compared among the four groups with Fisher’s exact test for categorical variables and the Kruskal-Wallis test for continuous variables. Numbers of samples in each cluster among four groups were compared using Fisher’s exact test. All four groups were statistically compared with beta-diversity analysis using permutational multivariate analysis of variance (PERMANOVA) to assess whether each microbiota of different groups is significantly different. Comparison of the relative abundance of *Lactobacillus* and the average episodes of cystitis per year between before and during administration of *Lactobacillus*-containing vaginal suppositories were performed by Mann–Whitney *U*-test and unpaired *t*-test, respectively. Results with *p* values <0.05 were considered statistically significant.

## Results

A total of 129 samples were analyzed from all 39 participants (Supplementary Figure 1). Nineteen participants with 38 samples were included from the healthy group. Twelve patients with 24 samples were included from the uncomplicated cystitis group. Among 8 patients with RC, 4 samples from 4 patients had not taken before administration of vaginal suppositories. Sixty-three samples were taken during follow-up period of administration of vaginal suppositories. Fourteen samples were regarded as the RC group, including 4 samples before administration of vaginal suppositories from 4 patients with RC, and 10 samples at the timing of the recurrence of RC from 5 patients among 8 patients during prevention. Among the 10 samples with RC during prevention, 7 samples from 4 patients among 5 patients were the same 4 patients with RC who were taken a sample, respectively, before administration of suppository. Except the 4 patients above, there were three samples from another patient who had the recurrence of RC during prevention, therefore totally 5 patients in the RC group were overlapped with those in prevention group. Fifty-three samples from eight patients were included from the prevention group, with no episodes of RC. There were no significant differences in patient characteristics among the groups (Supplementary Table 1).

**Figure 1:**
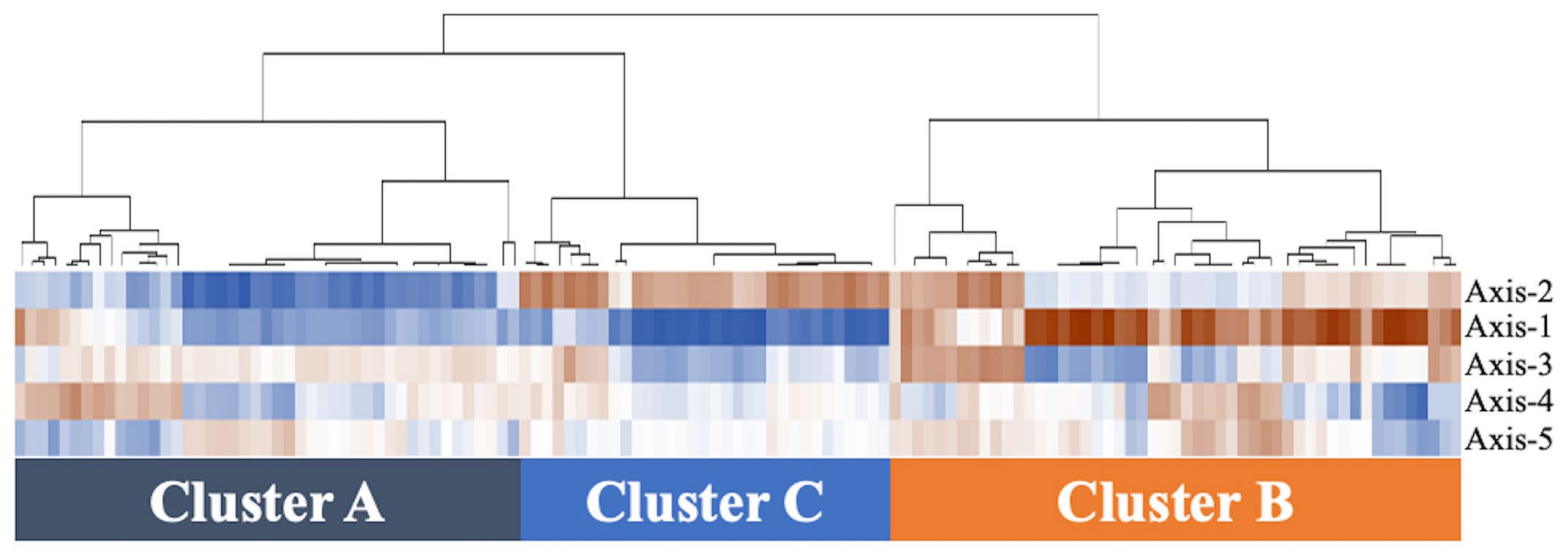
Hierarchical clustering analysis of vaginal microbiomes in 129 samples. The data from axes 1–5 of PCoA are included on the vertical axis. The dendrogram created is based on the correlation coefficient calculated by copy numbers between samples in 4 groups. The more vivid brown color represents the higher correlation coefficient (closer to 1), and the more vivid blue color represents the lower value of the correlation coefficient (closer to −1).

**Figure 2:**
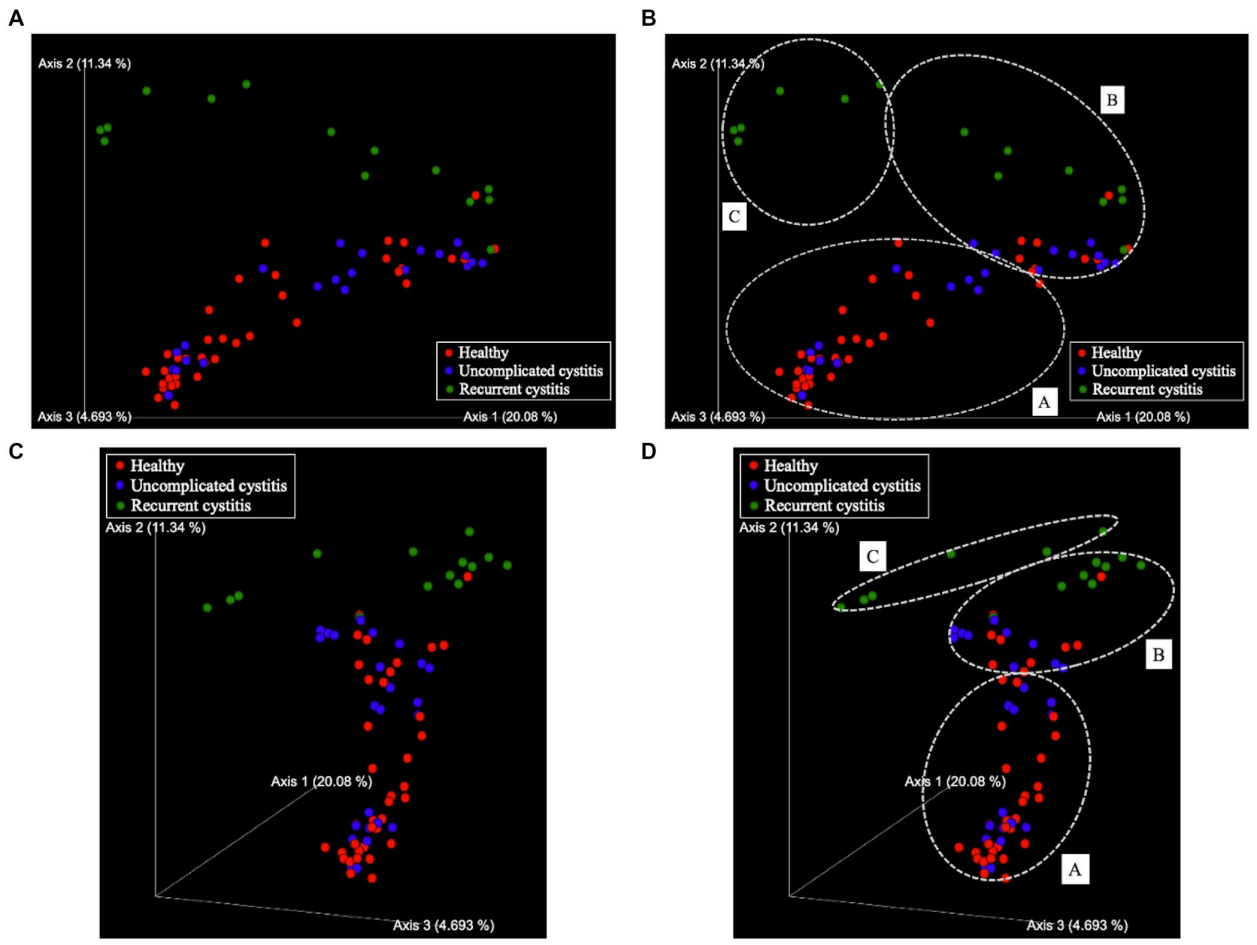
Principal coordinate analysis (PCoA). **(A)** Two-dimensional plots (except axis 3), **(B)** Three-dimensional plots. Samples are represented by dots. The three axes explain 20.08, 11.34, and 4.693% of the total variation. Red sphere: healthy, 38 samples in total; blue sphere: uncomplicated cystitis, 24 samples in total; green sphere: recurrent cystitis, 14 samples in total. **(C)** Clusters in two-dimensional plots. **(D)** Clusters in three-dimensional plots. Dashed white circles: clusters **(A–C)**.

**Figure 3:**
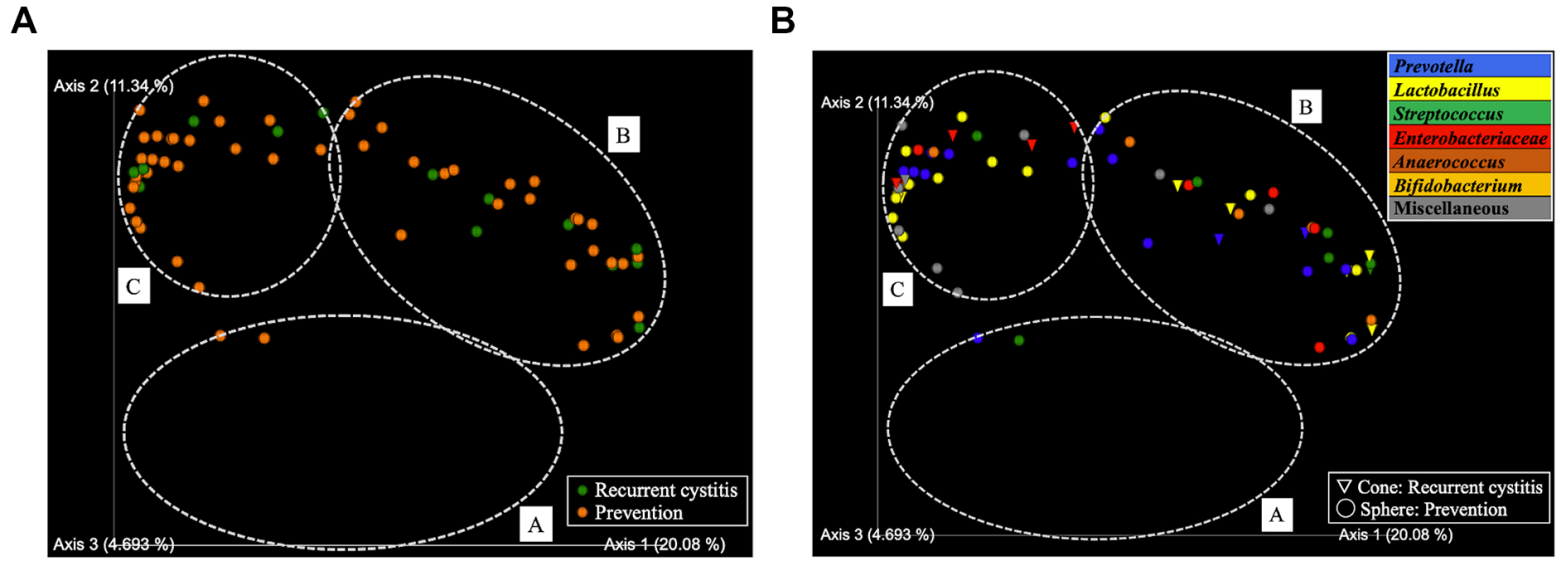
PCoA in two-dimensional plots among the recurrent cystitis group and prevention group. **(A)** Clusters. Green sphere: recurrent cystitis, 14 samples in total; orange sphere: prevention, 53 samples in total. **(B)** Predominant taxa. Cone: recurrent cystitis; sphere: prevention. Blue: *Prevotella*, yellow: *Lactobacillus*, green: *Streptococcus*, red: *Enterobacteriaceae*, brown: *Anaerococcus*, orange: *Bifidobacterium*, and gray: miscellaneous taxa.

Based on hierarchical cluster analysis, all 129 samples were divided into three distinct clusters (A, B, and C; Figure 1). Based on PCoA, the plots representing each sample and clusters A, B, and C are shown as two-and three-dimensional plots (Figures 2, 3). Among the 4 groups of women, 29 of 38 (76%) samples from the healthy group were in cluster A, 14 of 24 (58%) samples from the uncomplicated cystitis group were in cluster A, 14 of 14 (100%) samples from the RC group were in clusters B and C, and 51 of 53 (96%) samples from the prevention group were in clusters B and C (Supplementary Table 2). As shown in Figure 2, among the healthy, uncomplicated cystitis, and RC group plots, the RC plots are mostly classified in clusters B and C, whereas the healthy and uncomplicated cystitis plots are mainly in cluster A. The uncomplicated cystitis plots are similar to the healthy plots, and the RC plots are different from the uncomplicated cystitis plots. Figures 3 and Supplementary Figure 2 show the RC and prevention plots. The prevention plots are similar to the RC plots (Figure 3A). Based on beta-diversity analysis of PCoA, it seems that there were significant differences between the healthy group and the uncomplicated cystitis group (*p* = 0.045), and between the RC group and the uncomplicated cystitis group or the healthy group (*p* = 0.001, *p* = 0.001, respectively). There were no significant differences between the RC group and the prevention group (*p* = 0.446). PCoA demonstrated a large separation between the RC and healthy or uncomplicated cystitis group plots, and the vaginal microbiota in the RC group did not change appreciably between clusters B and C during prevention. Based on the relative abundance of bacterial taxa in the vaginal samples from each group, taxa with >10% abundance in at least one sample were identified, as shown in Supplementary Tables 3–6. The top six taxa are listed and colored differently in Figure 3B. The top six taxa were as follows: *Prevotella*; *Lactobacillus*; *Streptococcus*; *Enterobacteriaceae*; *Anaerococcus*; and *Bifidobacterium*. In cluster C, *Enterobacteriaceae* was mainly detected as the predominant taxon in RC plots, while *Lactobacillus* was detected as the predominant taxon in prevention plots.

**Figure 4:**
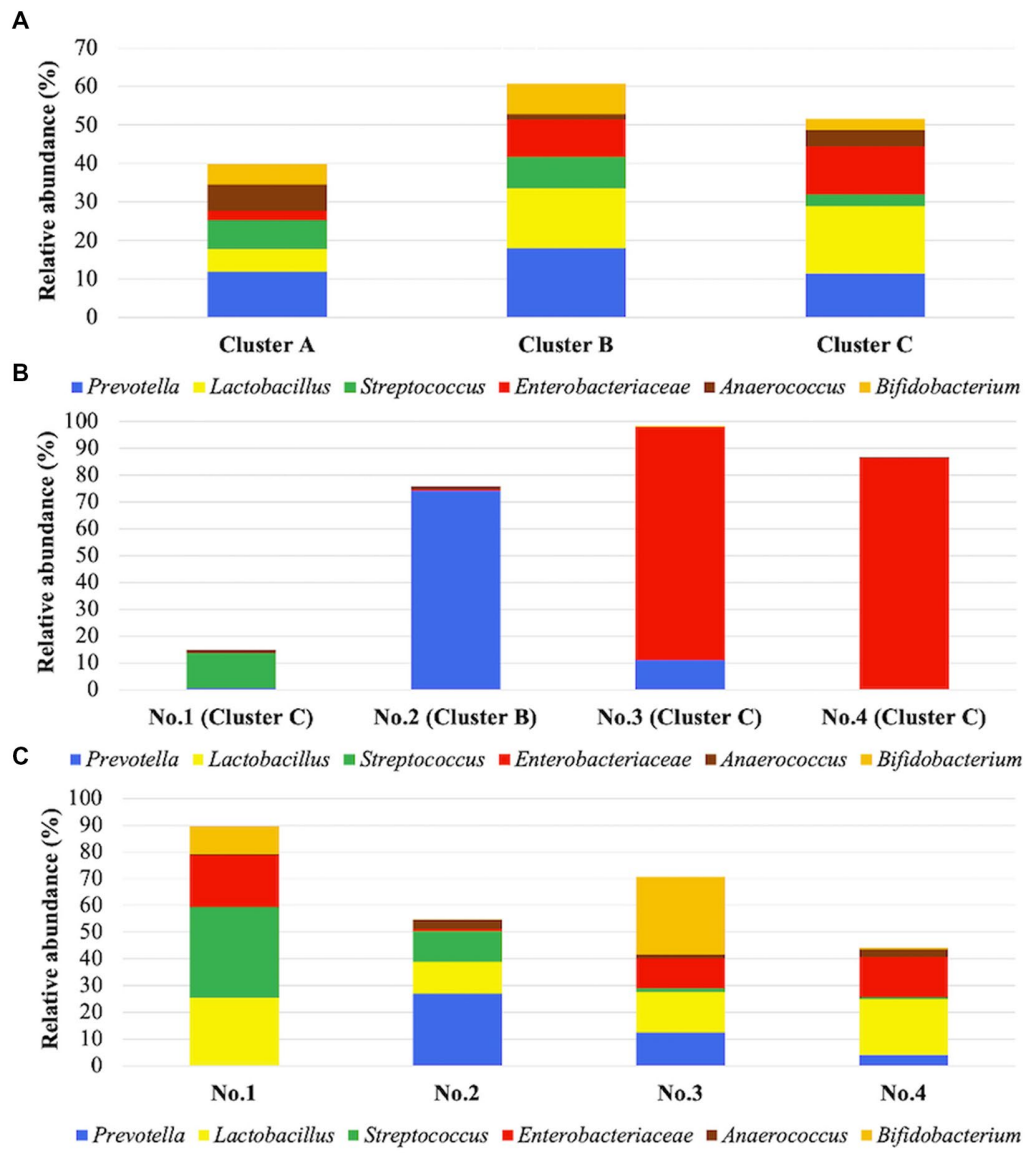
**(A)** Predominant taxa in each cluster. Cluster **(A)** includes 45 samples, cluster **(B)** includes 51 samples, and cluster C includes 33 samples. Stacked bars show the relative abundance of predominant taxa on average in each cluster. These six taxa were the most frequent among the taxa, comprising >10% of communities in at least one cluster. **(B)** Relative abundances in 4 patients before administration of suppositories. The four samples were from patients 1–4, respectively, in the recurrent cystitis group. **(C)** Relative abundances on average in 4 patients during administration of suppositories. The 53 samples were from patients 1–4 in the prevention group. Patients1–4 are shown on the horizontal axis. Stacked bars show the relative abundance of the six dominant taxa on average in each cluster, as also shown in **(B)**.

**Figure 5:**
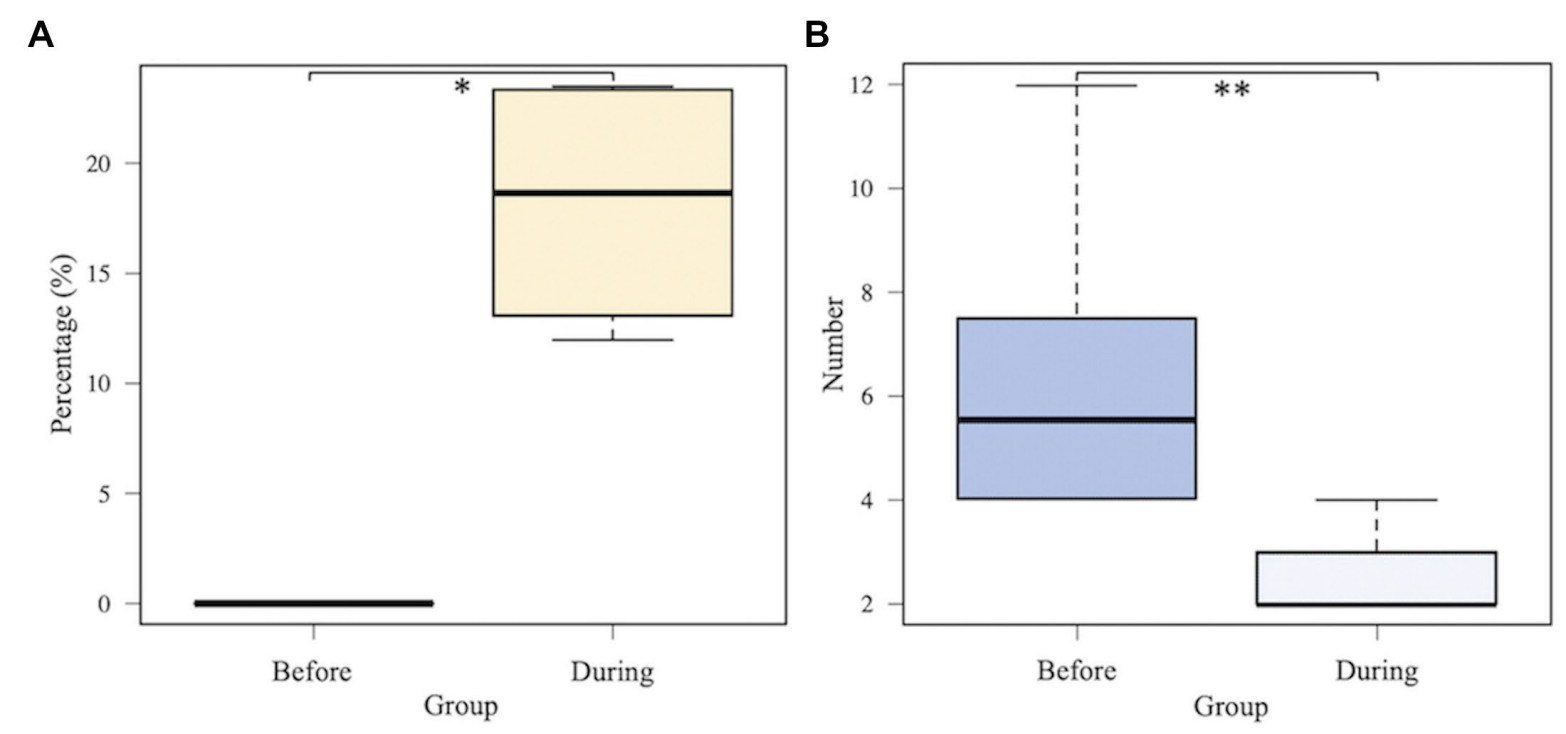
Comparison between before and during administration of *Lactobacillus-*containing vaginal suppositories. **(A)** The median (range) relative abundance of *Lactobacillus*. *Lactobacillus* was not detected before administration based on the 4 samples in patients 1–4 in the recurrent cystitis group; however, the median (range) relative abundance of *Lactobacillus* was 19% (14–23%) during administration based on the 36 samples in patients 1–4 in the prevention group using Mann–Whitney *U*-test (*p* = 0.0211). **(B)** Total number of episodes of recurrent cystitis per year. Eight patients in the prevention group had an average of 6.3 RC episodes per year before prevention, and an average of 2.4 RC episodes per year during prevention using unpaired *t*-test (*p* = 0.0015).

Considering the predominant six taxa in all samples, the relative abundances on average in each cluster are shown in Figure 4A. *Lactobacillus* was identified in clusters B and C with >15% relative abundance. In Figures 4B,C, the relative abundance of *Lactobacillus* in four patients was compared before and during administration of suppositories. In patients 1–4, *Lactobacillus* was not detected before administration based on the samples in the recurrent cystitis group; however, the median (range) relative abundance of *Lactobacillus* was 19% (14–23%) during administration based on the samples in the prevention group using Mann–Whitney *U*-test (*p* = 0.0211; Figure 5A). Eight patients in the prevention group had an average of 6.3 RC episodes per year before prevention with *Lactobacillus*-containing vaginal suppositories, and an average of 2.4 RC episodes per year during prevention using unpaired *t*-test (*p* = 0.0015; Figure 5B), showing the decrease in episodes of cystitis during prevention.

## Discussion

Using hierarchical cluster analysis, the vaginal bacterial communities were divided into three clusters based on differences in bacterial taxa among samples from each group. We showed that the vaginal microbiota in postmenopausal women with RC was different from the vaginal microbiota in postmenopausal women with uncomplicated cystitis or healthy controls, and vaginal dysbiosis can be associated with the cause of RC. This difference is caused by lack of *Lactobacillus* and relatively dominant of *Enterobacteriaceae* in the vaginal community. We also showed that a lack of *Lactobacillus* in postmenopausal vaginal microbiota can be associated with the onset of RC. Indeed, *Lactobacillus* supplementation of the vaginal microbiota reduced RC.

In this study, PCoA demonstrated a large separation between the RC and healthy or uncomplicated cystitis group plots. Differences in the microbiome from the urogenital tract between patients with RC and healthy women, and RC and uncomplicated cystitis have been reported (Stamey, 1973; Schaeffer and Stamey, 1977; Yoo et al., 2021). Our study corroborated the differences in the vaginal microbiome based on 16S rRNA sequencing analysis. PCoA also revealed the vaginal microbiota in the RC group did not change appreciably between clusters B and C during prevention. *Enterobacteriaceae* was predominant in the vaginal flora during RC episodes, especially in cluster C, and was stabilized by *Lactobacillus* during prevention. It is conceivable that *Lactobacilli* are less abundant in the vaginal microbiome of postmenopausal women (Zhang et al., 2012; Gliniewicz et al., 2019). Of note, we demonstrated that *Lactobacillus* colonization was restored during prevention, although *Lactobacillus* was not detected before prevention. *Lactobacillus* can produce several antimicrobial compounds and can form cell aggregates (Yungareva and Urshev, 2018), and *L. crispatus* can inhibit the growth of cystitis-associated, uropathogenic *E. coli* (UPEC) strains by decreasing their adhesion to the vaginal epithelial cells and by attenuating UPEC cytotoxic activity (Butler et al., 2016). Taken together with the decrease in episodes of cystitis during prevention, it is possible that UPEC could still be present in the vaginal microbiota but their virulence is inhibited following recolonization of *Lactobacillus*. We demonstrated that specific postmenopausal vaginal microbiotas are responsible for RC that can be stabilized with the vaginal administration of *Lactobacillus*. These findings indicate that vaginal dysbiosis can be associated with RC and continuous administration of *Lactobacillus*-containing suppositories can reduce RC. The link between decreasing RC and the use of *Lactobacillus*-containing vaginal suppositories has been indicated in another report, and we need to evaluate antimicrobial properties to counteract the targeted pathogens in vagina to develop innovative therapeutic strategies based on *Lactobacillus* strains (Atassi et al., 2019).

Moreover, the vaginal microbiota in postmenopausal women with RC did not return to the normal microbiota of healthy controls during intravaginal administration of *Lactobacillus*-containing suppositories. Nevertheless, the number of RC episodes decreased during intravaginal administration of *Lactobacillus*-containing suppositories, indicating the therapeutic effect for RC. *Lactobacillus* has several putative mechanisms by which vaginal colonization of uropathogens is prevented and the vaginal microbiota is stabilized. First, *Lactobacillus* produces biosurfactants and antimicrobial compounds, such as hydrogen peroxide (H_2_O_2_), lactic acid, and bacteriocins (McGroarty et al., 1992; Aroutcheva et al., 2001; Vallor et al., 2001; Tachedjian et al., 2017). Second, an important characteristic of *Lactobacillus* is blockage of the adherence and growth of uropathogens by interference with adhesion receptors or coaggregation with pathogens, achieving a healthy state in the vagina (Boris et al., 1998; Reid et al., 1998; Osset et al., 2001; Chee et al., 2020). These mechanisms are considered to explain, at least in part, how *Lactobacillus* exerts beneficial effects on the vaginal microbiota.

Importantly, our data suggest that non-antibacterial prophylaxis can be considered for patients with RC by the vaginal administration of *Lactobacillus*. Additionally, some vaccinations to prevent RC have been recently evaluated. A sublingual bacterial preparation of inactivated whole-cell bacteria has shown clinical efficacy (Lorenzo-Gómez et al., 2022). This effect can be achieved by antibody production and activation of dendritic cells to induce anti-inflammatory T-cell generation and responses in the bladder and secondary lymphoid organs (Benito-Villalvilla et al., 2017; Martin-Cruz et al., 2021). Vaginal administration of *Lactobacillus*, in contrast, has clinical efficacy by improving the vaginal microbiota, which could function by reducing the virulence of uropathogenic bacteria in the vagina and also could reduce the abundance of uropathogenic bacteria in the vagina which in turn could help limit migration from the vagina to the bladder. *L. crispatus* GAI 98322-containing vaginal suppositories can be an adjunct to vaccination as a non-antibiotic alternative for the prevention of RC.

The present study had some limitations. First, the study had the small numbers of patients and samples, and a retrospective cohort design. Among 8 patients with RC, 4 samples from 4 patients before administration of vaginal suppositories had not taken, and patients in RC group were overlapped with those in prevention group. However, no long-term data have been reported on the vaginal microbiota in patients with or without RC prevented by vaginal suppositories. It is noteworthy that we included patients with more frequent episodes of cystitis than the minimum number of 3 episodes of cystitis per year by definition, estimated as severe cases with RC. Our study showed that *L. crispatus* GAI 98322-containing vaginal suppositories were effective in decreasing the number of RC episodes by 62% among 8 patients. Our previous study also demonstrated that *L. crispatus* GAI 98322-containing vaginal suppositories decreased the number of RC episodes by >70% in 18 of 21 patients (Sadahira et al., 2021). Despite the small number of patients, this study demonstrated that even postmenopausal women with refractory RC with average 6.3 episodes of cystitis per year responded to the administration of vaginal suppositories containing *Lactobacillus.* Although analysis with a larger number of samples will be necessary in the future, there are not so many cases with RC in general, and there are few reports of such analyses among these relatively rare patients. We believe that the result in this study is clinically important and the results of this study are also valid, as shown the data from unpaired *t*-test or beta-diversity analysis of PCoA among the overall sample size of 129. Second, some bacterial taxa were included as an unknown genus or species in the data obtained from vaginal microbiomes; however, we focused specifically on *Lactobacillus*. Therefore, another unknown genus or species had less effect on the conclusions. Third, although recent reports assessed the relationship between urinary or gut dysbiosis and RC (Yoo et al., 2021; Worby et al., 2022), we did not assess the urinary or gut microbiota and only focused on vaginal microbiota in this study. Conducting a combined analysis of these three types of microbiotas will be essential to establish more evidence between dysbiosis and RC. Lastly, repeated antibiotic exposure itself may cause dysbiosis. Further long-term studies are needed to determine the impact of antibiotics to affect the vaginal microbiota.

In conclusion, the present study demonstrated that the vaginal microbiota in postmenopausal women with RC is essentially different from that of postmenopausal women with uncomplicated cystitis. Therefore, vaginal dysbiosis is likely associated with RC because the vagina serves as a reservoir of enteric bacteria, from which cystitis becomes intractable. The administration of vaginal suppositories containing *Lactobacillus* has a suppressive effect for preventing RC.

## Data availability statement

The datasets presented in this study can be found in online repositories. The names of the repository/repositories and accession number(s) can be found in the article/Supplementary material.

## Ethics statement

The studies involving human participants were reviewed and approved by Okayama University Hospital. The patients/participants provided their written informed consent to participate in this study.

## Author contributions

TSe and TSa interpreted the results and drafted the manuscript. KW, TSa, and AI was involved in sample collection and conducted the experimental work. KW, TI, MW, and MA supervised the experiments and were involved in editing the manuscript. TM and ST performed analysis of the microbiome data. MA performed English editing of the manuscript. MW and TSa supervised the study and performed a critical review of the manuscript. All authors read and approved the final manuscript.

## Funding

This study was supported by a scientific research grant from the Ministry of Education, Culture, Sports, Science and Technology of Japan (JSPS KAKENHI grant numbers: JP17K11183 and JP20K18117), and by a scientific research grant from the Japanese Society of Chemotherapy Foundation (Uehara Infectious Disease and Chemotherapy Research Award), and by GSK Japan Research Grant 2017.

## Conflict of interest

The authors declare that the research was conducted in the absence of any commercial or financial relationships that could be construed as a potential conflict of interest.

## Publisher’s note

All claims expressed in this article are solely those of the authors and do not necessarily represent those of their affiliated organizations, or those of the publisher, the editors and the reviewers. Any product that may be evaluated in this article, or claim that may be made by its manufacturer, is not guaranteed or endorsed by the publisher.
