# Supplementary figures and images for: Etiology of recurrent cystitis in postmenopausal women based on vaginal microbiota and the role of Lactobacillus vaginal suppository
Source: Front Microbiol. 2023 May 18;14:1187479. doi: 10.3389/fmicb.2023.1187479 (PMC10232810; doi:10.3389/fmicb.2023.1187479)

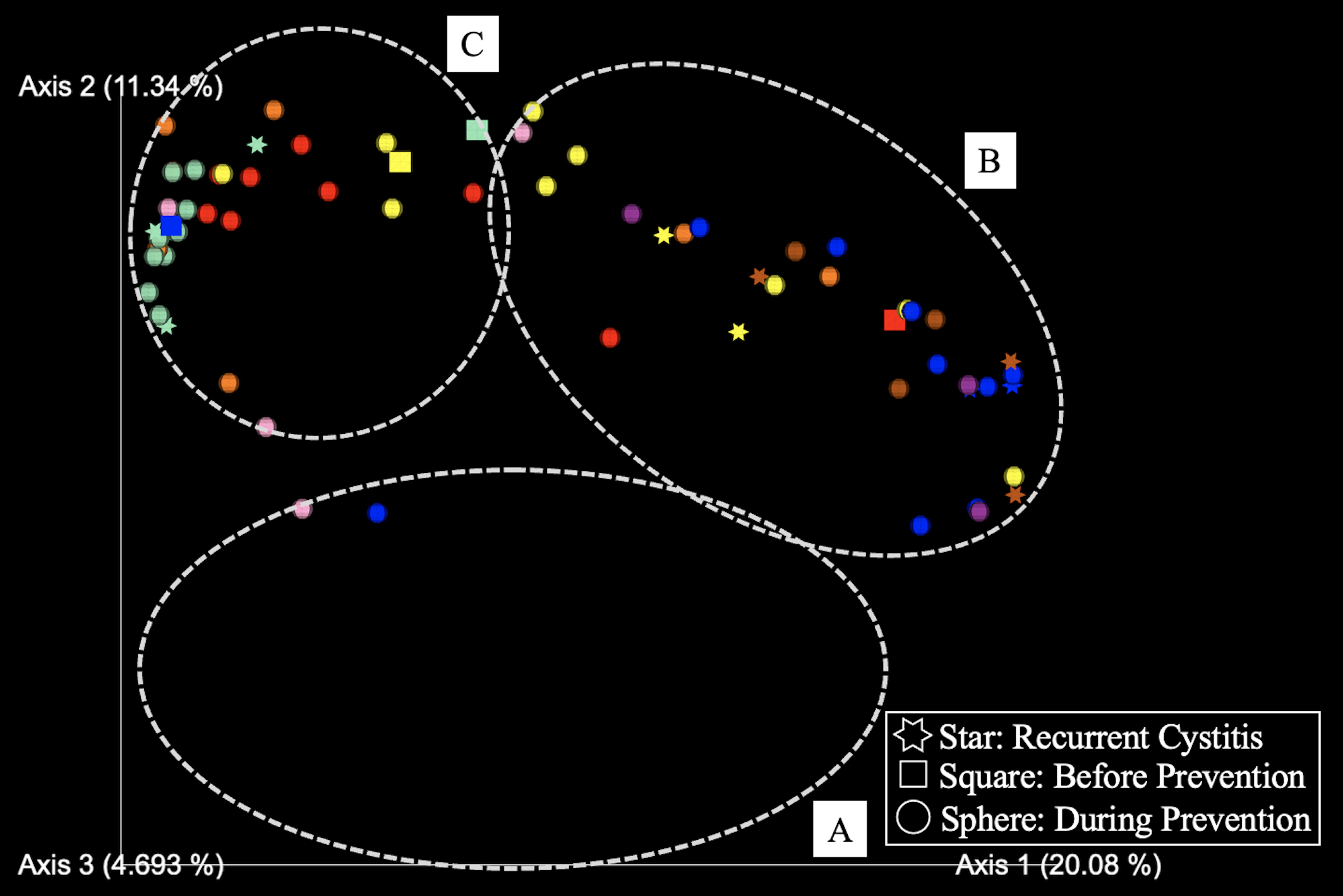

Supplement: Supplementary file 1 [file Image_1.JPEG]

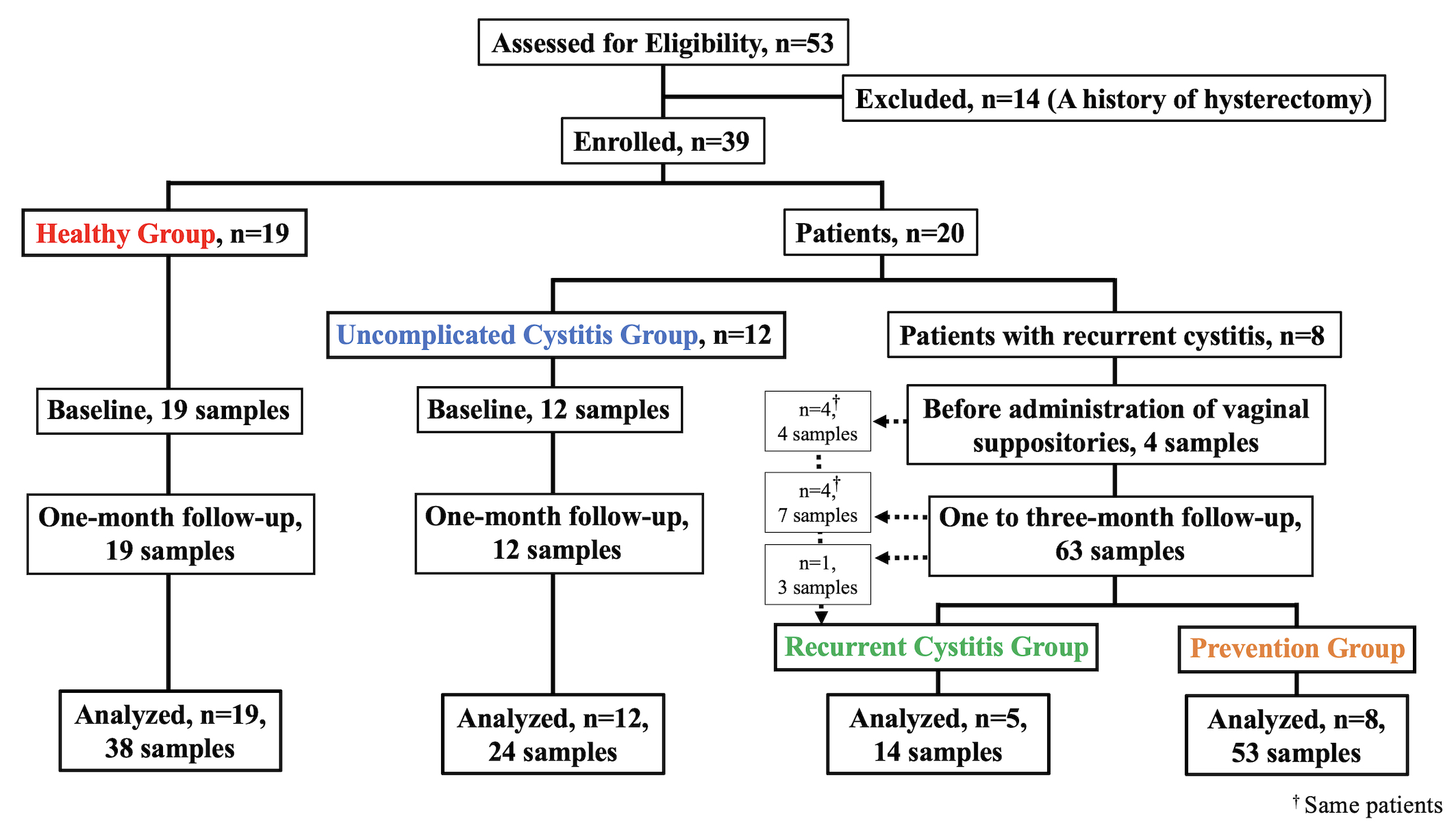

Supplement: Supplementary file 2 [file Image_2.JPEG]
