# Supplementary material for: Etiology of recurrent cystitis in postmenopausal women based on vaginal microbiota and the role of Lactobacillus vaginal suppository
Source: Front Microbiol. 2023 May 18;14:1187479. doi: 10.3389/fmicb.2023.1187479 (PMC10232810; doi:10.3389/fmicb.2023.1187479)
Supplement: Supplementary file 3 [file Table_1.DOCX]

**Supplementary Table 1. Patient characteristics**

IQR; interquartile range, BMI; body mass index, DM; diabetes mellitus, NB; neurogenic bladder

^†^ Including 5 patients in Recurrent cystitis

|  | Total | Healthy | Uncomplicated cystitis | Recurrent cystitis | Prevention | *p* |
| --- | --- | --- | --- | --- | --- | --- |
| Total cases, n (%) | 39 | 19 | 12 | 5 | 8^†^ |  |
| Age, median (IQR) | 73.0 (66.0-78.0) | 73.0 (64.5-78.0) | 72.5 (64.3-78.5) | 70.0 (66.0-73.0) | 71.5 (66.0-74.0) | 0.639 |
| BMI, median (IQR) | 23.0 (21.3-26.6) | 22.6 (20.6-27.0) | 23.7 (21.4-27.0) | 23.1 (22.9-24.2) | 23.3 (23.0-24.0) | 0.813 |
| DM, n (%) | 10 (25.6) | 4 (21.1) | 3 (25.0) | 1 (20.0) | 2 (25.0) | 1.000 |
| NB, n (%) | 1 (2.6) | 0 (0.0) | 1 (8.3) | 0 (0.0) | 0 (0.0) | 0.568 |

**Supplementary Table 2. Numbers of samples in each cluster among 4 groups.**

|  | Healthy | Uncomplicated cystitis | Recurrent cystitis | Prevention |
| --- | --- | --- | --- | --- |
| Cluster A | 29 | 14 | 0 | 2 |
| Cluster B | 9 | 10 | 8 | 24 |
| Cluster C | 0 | 0 | 6 | 27 |
| Total | 38 | 24 | 14 | 53 |

*p* < 0.001

**Supplementary Table 3. Relative abundance of bacterial taxa in vaginal samples obtained from the “Healthy” group.**

No.4

No.8

No.4

**
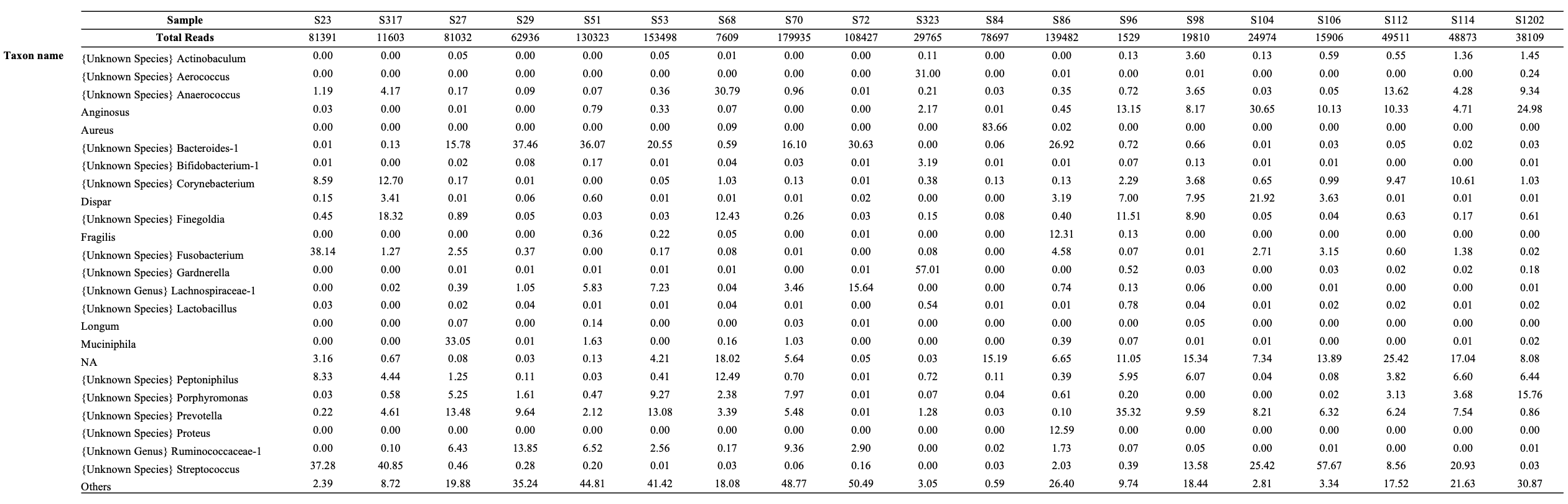
Supplementary Table 3-1.**

Taxa with >10% abundance in at least one sample are included. Taxa in the samples with < 10% abundance in all samples are aggregated into category: "Others.” NA: not applicable


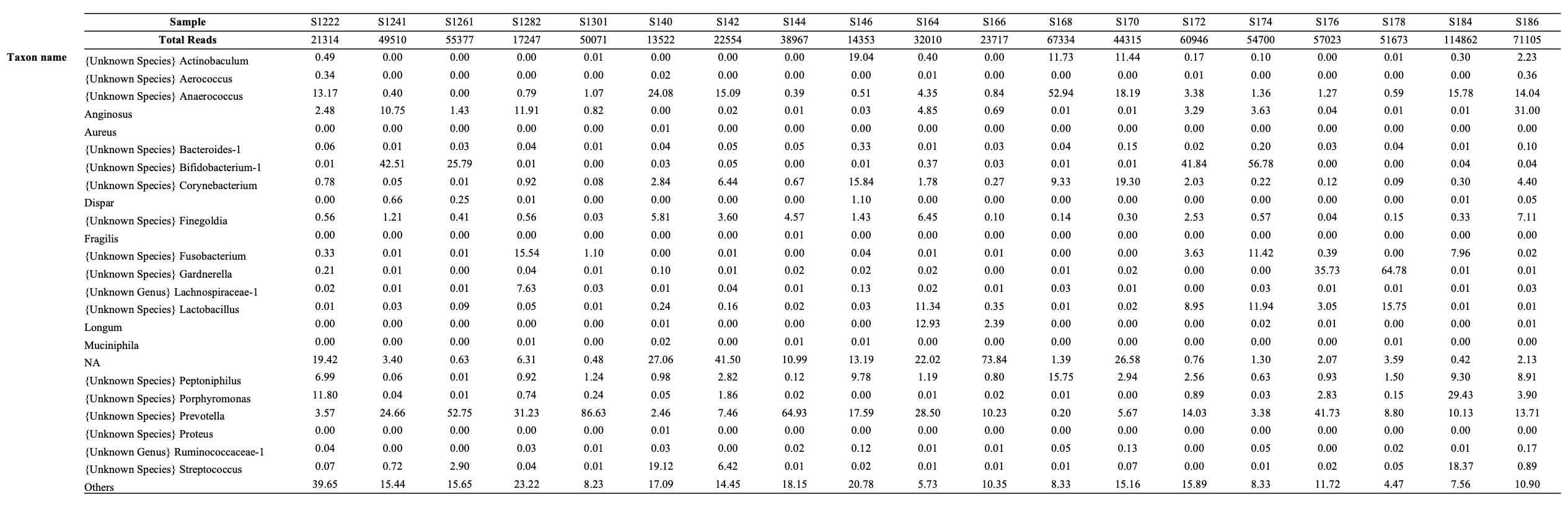
**Supplementary Table 3-2.**

Taxa with >10% abundance in at least one sample are included. Taxa in the samples with < 10% abundance in all samples are aggregated into category: "Others.” NA: not applicable

**
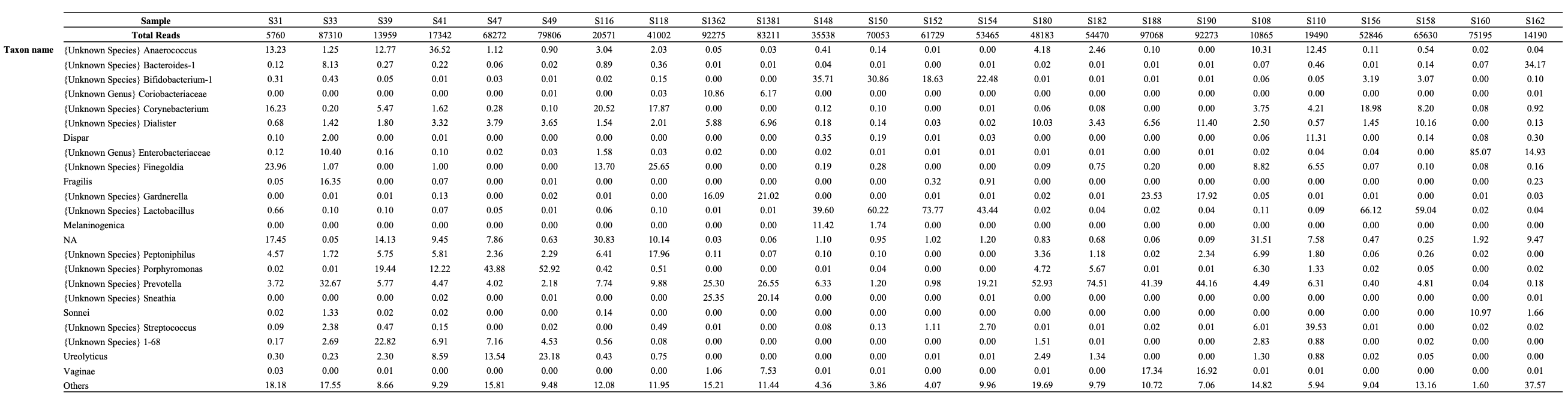
Supplementary Table 4. Relative abundance of bacterial taxa in vaginal samples obtained from “Uncomplicated cystitis” group**

Taxa with >10% abundance in at least one sample are included. Taxa in the samples with < 10% abundance in all samples are aggregated into category: "Others.” NA: not applicable

**
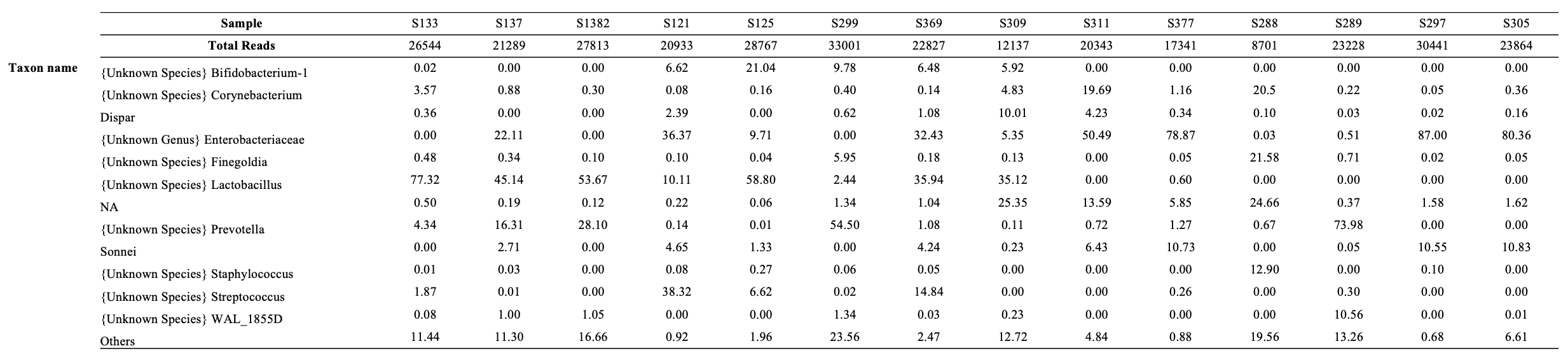
Supplementary Table 5. Relative abundance of bacterial taxa in vaginal samples obtained from “Recurrent cystitis” group**

Taxa with >10% abundance in at least one sample are included. Taxa in the samples with < 10% abundance in all samples are aggregated into category: "Others.” NA: not applicable

**Supplementary Table 6. Relative abundance of bacterial taxa in vaginal samples obtained from “Prevention” group**

**
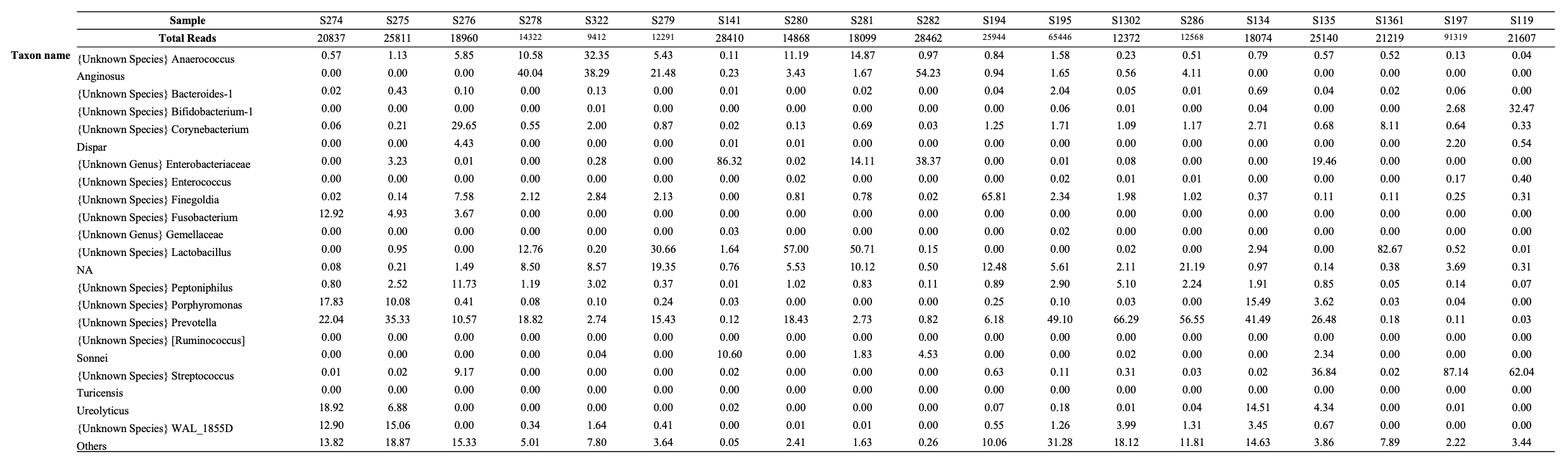
Supplementary Table 6-1.**

Taxa with >10% abundance in at least one sample are included. Taxa in the samples with < 10% abundance in all samples are aggregated into category: "Others.” NA: not applicable

**
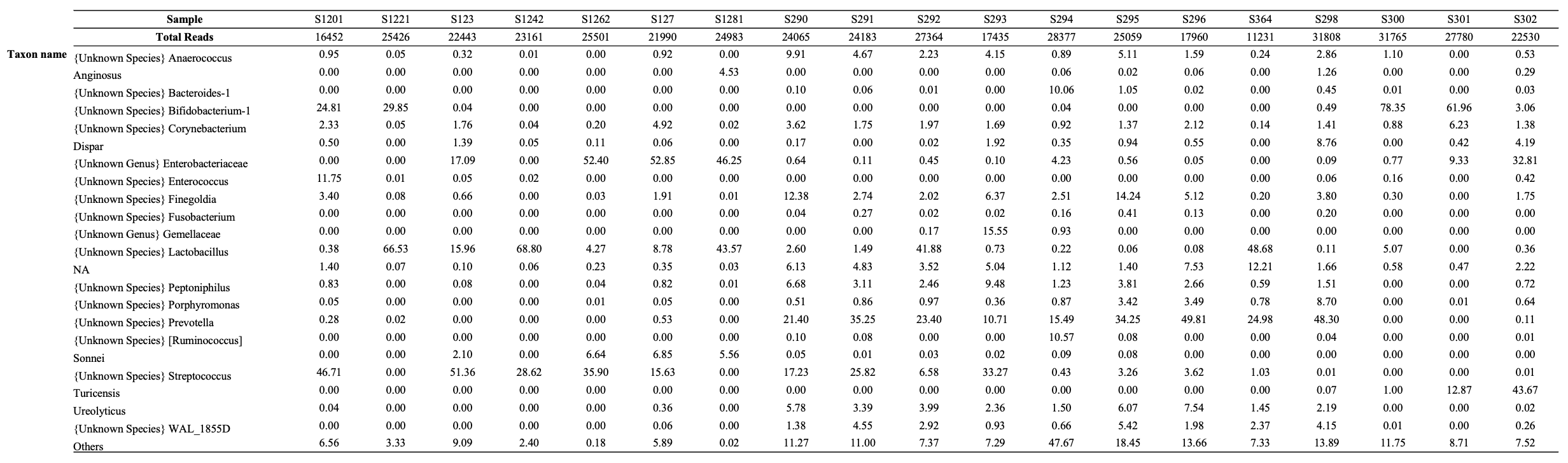
Supplementary Table 6-2.**

Taxa with >10% abundance in at least one sample are included. Taxa in the samples with < 10% abundance in all samples are aggregated into category: "Others.” NA: not applicable

**
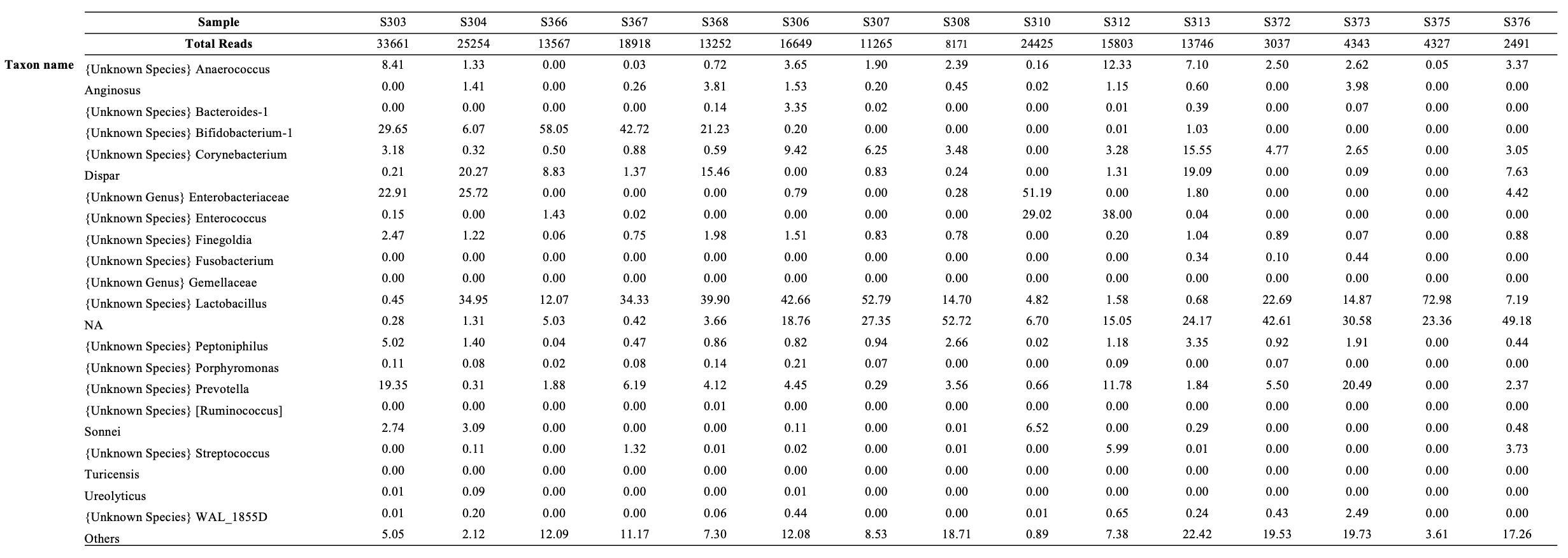
Supplementary Table 6-3.**

Taxa with >10% abundance in at least one sample are included. Taxa in the samples with < 10% abundance in all samples are aggregated into category: "Others.” NA: not applicable

**Supplementary Figure 1. Flow diagram of study selection process and the total number of follow-up participants and samples.** Nineteen participants with 38 samples were included from the healthy group. Twelve patients with 24 samples were included from the uncomplicated cystitis group. Among 8 patients with RC, 4 samples from 4 patients before administration of vaginal suppositories had not taken. Sixty-three samples were taken during follow-up period of administration of vaginal suppositories. Fourteen samples were regarded as the RC group, including 4 samples before administration of vaginal suppositories from 4 patients with RC, and 10 samples at the timing of the recurrence of RC from 5 patients among 8 patients during prevention. Among the 10 samples with RC during prevention, 7 samples from 4 patients among 5 patients were the same 4 patients with RC who were taken a sample respectively before administration of suppository. Except the 4 patients above, there were three samples from another patient who had the recurrence of RC during prevention, therefore totally 5 patients in the RC group were overlapped with those in prevention group. Fifty-three samples from eight patients were included from the prevention group, with no episodes of RC. RC: Recurrent Cystitis.

**Supplementary Figure 2: Principal coordinate analysis (PCoA) in two-dimensional plots** **among the recurrent cystitis group and prevention group.** Totally 67 samples among 8 patients in the recurrent cystitis group and prevention group are represented by dots. Samples from the same individuals have the same color. Star: RC; square: samples with RC before prevention; sphere: samples during prevention without RC. RC: Recurrent Cystitis.
